# Long-term outcome after chronic anastomotic leakage following surgery for low rectal cancer

**DOI:** 10.1007/s00384-022-04213-8

**Published:** 2022-07-12

**Authors:** Florian Ponholzer, Clemens Paul Klingler, Elisabeth Gasser, Philipp Gehwolf, Marijana Ninkovic, Ruben Bellotti, Reinhold Kafka-Ritsch, Dietmar Öfner

**Affiliations:** Department of Visceral, Transplant and Thoracic Surgery, Center of Operative Medicine, Medical University of Innsbruck, Anichstrasse 35, 6020 Innsbruck, Austria

**Keywords:** Low rectal cancer, Chronic anastomotic leakage, Chronic presacral sinus, Bowel continuity

## Abstract

**Purpose:**

This study analyzed the prevalence and factors influencing the history of chronic anastomotic leakage following low anterior resection for rectal cancer. Furthermore, the treatment of a persisting presacral sinus and the impact of stoma reversal on outcome were evaluated.

**Methods:**

The institutional database was scanned for all patients with anastomotic leakage, who were primarily treated for low rectal cancer between January 1995 and December 2019. Patients with rectovaginal and rectovesical fistula or an inadequate follow-up were excluded (*n* = 5). After applying the exclusion criteria, 71 patients remained for analysis.

**Results:**

A total of 39 patients out of 71 patients with anastomotic leakage (*54.9%*) developed a persisting presacral sinus. Neoadjuvant radiochemotherapy or chemotherapy showed a significant impact on the formation of a chronic anastomotic leakage (radiochemotherapy: *p* = 0.034; chemotherapy: *p* = 0.050), while initial surgical treatment showed no difference for anastomotic healing (*p* = 0.502), but a significantly better overall survival (*p* = 0.042). Multiple therapies and surgical revision had a negative impact on patients’ rate of natural bowel continuity (*p* = 0.006/ < 0.001). In addition, the stoma reversal cohort showed improved overall 10-year survival (*p* = 0.004) and functional results (bowel continuity: *p* = 0.026; pain: *p* = 0.031).

**Conclusion:**

Primary surgical therapy for chronic anastomotic leakage should consist of surgical treatment. Furthermore, the reversal of a protective stoma should be considered a viable option in treating chronic presacral sinus to improve pain symptoms and bowel continuity.

## Introduction

Colorectal cancer represents the third most common malignant tumor worldwide [1]. After surgical treatment, acute anastomotic leakage (AL) is the most feared postoperative complication, with an incidence of up to 20% in rectal cancer, increasing patients’ morbidity and mortality by leading to worse oncological and functional outcomes and formation of chronic anastomotic leakage (CAL) or a persisting presacral sinus, which is associated with further complications [2–6].

Surgical treatment of low rectal cancer and its early postoperative complications has become relatively standardized and is already thoroughly reported. Still, only limited literature is available for late-onset postoperative morbidity management, such as CAL. CAL represents a complication with complex treatment strategies and a severe impact on patients’ quality of life, some even requiring a permanent colostomy [7, 8]. These treatment strategies might also be impacted by reduced healing tendency, through increased inflammation in the affected area, in patients who received neoadjuvant radio- or chemotherapy [9].

AL and CAL may lead to systemic infections and impaired intestinal continuity, while CAL may especially result in the formation of a recurrent fistula or a persisting presacral sinus [10, 11]. Secondary complications may include involvement of the surrounding tissue, leading to osteomyelitis, necrotizing fasciitis, and periureteric fibrosis with ureteral stenosis and hydronephrosis [11]. Additionally, permanent inflammation in the region of the chronic sinus, together with the epithelialization of the surface, promotes increased cell proliferation and cell turnover rate, which increase the chance for further malignant transformations. However, since such de novo carcinomas are a rarity, extensive long-term observations would be necessary to demonstrate a connection between chronic sinus and malignant tumor development [12].

Accordingly, the literature regarding treatment strategies in patients with CAL is very heterogeneous.

In general, the treatment ranges from conservative over interventional/endoscopic to surgical management, with various individual pathways in each category. Because there is no thoroughly established treatment algorithm, some patients might also receive unnecessary multimodal treatment after primary therapy failure (e.g., permanent terminal stoma), resulting in further physical, psychological, and financial strain for these patients in some health care systems [3, 13, 14].

Furthermore, in hindsight of rising budgetary pressure on public health care systems, it seems only logical to implement a treatment algorithm for CAL with a persisting presacral sinus to reduce costs for long-term complication treatment and preventable treatment modalities.

This study aims to analyze the prevalence of CAL, suspected risk factors (e.g., chemo-/radiotherapy or surgical technique) for its development, the treatment modalities (conservative, interventional, or surgical) in patients who received surgery for low rectal cancer, and the possible influence of stoma reversal on postoperative pain and bowel continuity in patients with presacral sinus.

## Methods

### Patient selection

**Fig. 1:**
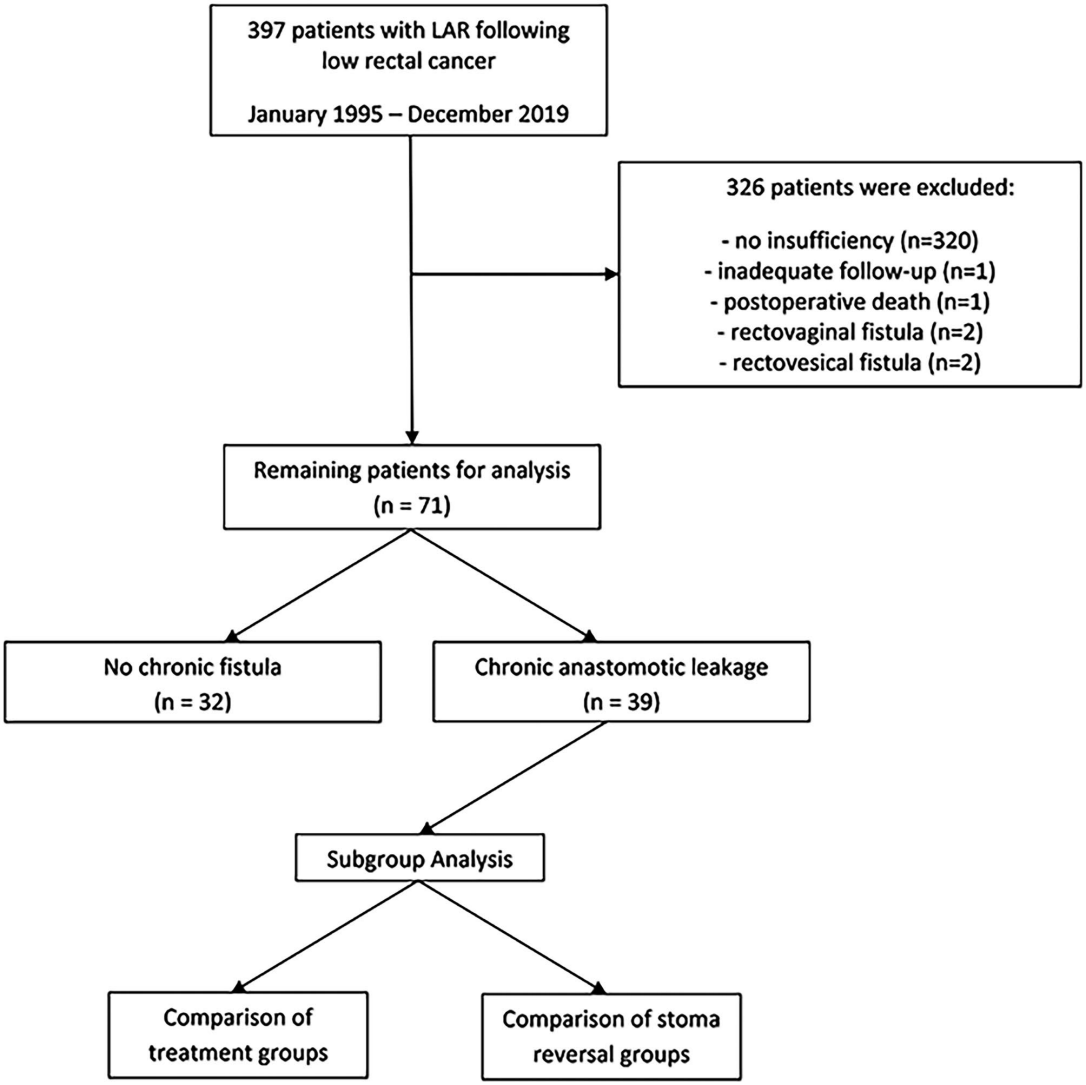
Flowchart of patient selection for statistical analysis

Before data collection, the local ethics committee granted the authorization for this retrospective single-center cohort study. Data from a total of 397 patients with low rectal cancer who were treated at the Department of Visceral, Transplant and Thoracic Surgery, Medical University of Innsbruck, between January 1995 and December 2019 were collected. Patients without anastomotic leakage, inadequate follow-up, postoperative death (cut-off 30th POD), and rectovesical or rectovaginal fistula were excluded. After applying the exclusion criteria, 71 patients with anastomotic leakage following rectal surgery remained for further statistical analysis. These 71 patients were split into two cohorts (AL vs. CAL) and compared, see Fig. 1.

### Data collection

All research was performed in accordance within national guidelines and regulations; the ethics committee waived individual informed consent due to the study design. Patients’ data were collected in a prospectively maintained database. Recorded data included but was not limited to patient history, oncological data, surgical data, data regarding the anastomotic leakage and its treatment, postoperative course, and patients’ survival.

### Study endpoints

The primary analysis of our study was to determine the prevalence of CAL and whether possibly associated factors for the occurrence of chronic insufficiency and the formation of a presacral sinus can be identified. A secondary analysis was performed for the CAL cohort to examine whether the initial treatment modality and reversal of a protective stoma have an association with the outcome regarding the healing of chronic anastomotic leakage.

### Definitions

An anastomotic leakage was defined according to Rahbari et al. “Defect of the intestinal wall integrity at the colorectal or colo-anal anastomotic site (including suture and staple lines of neorectal reservoirs) leading to a communication between the intra- and extraluminal compartments. A pelvic abscess close to the anastomosis is also considered as anastomotic leakage.” and was considered acute if it developed in the first 30 days after surgery [15].

Development of chronic anastomotic leakage was defined as persistence of acute AL and/or primary diagnosis of anastomotic dehiscence longer than 30 days after primary surgery. Presacral sinus formation was diagnosed following radiologic or endoscopic validation, which included descriptions of abscess formations, necrotic cavities, blindly ending fistulas, and with the intestinal lumen communicating retentions.

For the localization of the tumor, the rectum was generally defined, according to Salerno et al. “as composed of three parts: the low rectum (up to 6 cm from the anal verge), the mid rectum (from 7 to 11 cm) and the upper rectum (from 12 to 15 cm).” [16]. For large carcinomas which extended over several parts, the aboral third was noted. If information was only available on the distance between the lower edge of the tumor and the dentate line, 2 cm was added to receive the distance to the anal verge.

Surgical revision was defined as any surgical intervention requiring laparotomy or laparoscopy.

### Therapy of anastomotic dehiscence

#### Treatment concepts

For retrospective statistical analysis and comparison, treatment concepts were classified according to the standardized consensus definition and severity grading of the International Study Group of Rectal Cancer adapted by Rahbari et al. [15]:

- Grade A: Anastomotic leakage requiring no active therapeutic intervention
- Grade B: Anastomotic leakage requiring active therapeutic intervention but manageable without relaparotomy
- Grade C: Anastomotic leakage requiring re-laparotomy.

Therefore, the study population was divided into three therapy groups based on their initial treatment modality: conservative (group A), interventional (endoscopic or radiologic intervention; group B), and surgical (group C) management. In their further therapy course, these groups were analyzed regarding the need for multiple therapies (treatment that required more than one intervention) or surgical revision.

##### Therapy success

At the last follow-up, two target points were defined, e.g., at the outpatient department or control appointment. An anamnesis regarding intestinal symptoms was taken to compare the therapeutic success of the various treatment concepts.

- No complaints regarding pain as an indicator of possible post-therapeutic discomfort of the patient. The pain was defined as subjectively perceived complaints in the rectal, gluteal, and/or sacral region.
- Current bowel continuity and the possibility of a natural bowel passage without a temporary or permanent terminal stoma.

### Statistical analysis

Statistical analysis was performed with IBM SPSS-Statistics Software Version 26 (IBM Corporation; Armonk, NY, USA). In R the package “survminer’ was used for plotting survival curves [17].

To analyze overall survival, Kaplan–Meier estimator was used. To adapt to the prolonged observation period, we used both Breslow and log rank test to check for statistical significance and receive a more detailed interpretation. Chi-squared tests and Fisher’s exact test were performed to compare qualitative and categorical variables, while independent samples T-test, due to the small sample size and its robustness against possibly skewed data, was used to compare continuous variables. The Kruskal–Wallis test was carried out for ordinal variables and the one-way ANOVA for continuous variables for several independent samples. Odds ratio and confidence intervall for factors, which might influence the development of CAL or therapeutical outcome, were calculated using the chi-squared test. A post hoc Bonferroni correction followed this to adjust for multiple testing. Pairwise deletion was used for missing data. Statistical significance was assumed for a *p*-value ≤ 0.05.

## Results

### Patient demographics and prevalence of chronic anastomotic leakage

Between January 1995 and December 2019, 397 patients were surgically treated with a low anterior resection for low rectal cancer at the Department for Visceral, Transplant and Thoracic Surgery, Medical University of Innsbruck. Among these patients, 71 (9.8%) developed some form of anastomotic leakage, with 39 (54.9%) of these developing chronic anastomotic dehiscence. There was no difference in age, sex distribution, or T classification between cohorts (*p* = 0.650/1.000/0.457), as shown in Table 1.

**Table 1:** Patient demographics

Factor*		Total, *n* = 71	Acute anastomotic leakage, *n* = 32	Chronic anastomotic leakage, *n* = 39	*p*-value
Age in years, mean (median, range)		63.77 (65.0, 35–89)	64.44 (65.5, 35–88)	63.23 (64.0, 39–89)	0.650
Sex (%)	Female Male	17 (23.9) 54 (76.1)	8 (25.0) 24 (75.0)	9 (23.1) 30 (76.9)	1.000
Neoadjuvant therapy (%)					
nCTx	nCTx no nCTx	44 (62) 26 (36.6)	16 (50) 16 (50)	28 (73.7) 10 (26.3)	**0.050**
nRCTx	nRCTx no nRCTx	39 (54.9) 32 (45.1)	13 (40.6) 19 (59.4)	26 (66.7) 13 (33.3)	**0.034**
Distance from the anal verge (cm), mean (median, range)*		7.37 (7.0, 3–15)	7.61 (7.5, 4–15)	7.20 (6.0, 3–15)	0.674
Surgical technique (%)*	Hand sewn Stapled	4 (5.6) 57 (80.3)	2 (6.3) 23 (71.9)	2 (5.1) 34 (87.2)	1.000
Anastomotic type (%)*	E-E S-E	20 (28.2) 47 (66.2)	13 (40.6) 16 (50.0)	7 (17.9) 31 (79.5)	**0.030**
T-staging, T (%)*	2 3 4	5 (7.0) 47 (66.2) 5 (7.0)	3 (9.4) 21 (65.6) 1 (3.1)	2 (5.1) 26 (66.7) 4 (10.3)	0.457
Pathological UICC stage (%)	0 I II III IV	4 (5.6) 14 (19.7) 15 (21.1) 22 (31.0) 16 (22.5)	2 (6.3) 6 (18.8) 2 (6.3) 11 (34.4) 11 (34.4)	2 (5.1) 8 (20.5) 13 (33.3) 11 (28.2) 5 (12.8)	**0.029**
Pathological grading (%)*	0 1 2 3	1 (1.4) 4 (5.6) 60 (84.5) 6 (8.5)	0 (0) 1 (3.1) 28 (87.5) 3 (9.4)	1 (2.6) 3 (7.7) 32 (82.1) 3 (7.7)	0.822
Residual tumour classification (%)	0 1 2	68 (95.8) 0 (0.0) 2 (2.8)	30 (93.75) 0 (0.0) 1 (3.1)	38 (97.4) 0 (0.0) 1 (2.6)	1.000

*Some factors do not add up to 100% as data might have been inconclusive or unavailable

### Factors influencing the formation of CAL

An association of neoadjuvant chemotherapy only (50.0 vs. 73.7%, OR = 2.80; CI:1.03–7.62) and neoadjuvant radiochemotherapy (40.6 vs. 66.7%, OR = 2.92; CI:1.11–7.71) with the formation of a persistent presacral sinus could be seen. Adjuvant chemotherapy or radiotherapy did not show an association with the formation of CAL (55.2 vs. 52.6%, *p* = 1.000; 9.4 vs. 10.3%, *p* = 1.000). Comparing the surgical technique, the rate of end-to-end anastomosis was significantly lower in the CAL cohort with 17.9% (vs. 40.6%, OR = 3.6; CI:1.2–10.8), although the rate of hand-sewn anastomosis did not differ between cohorts.

### Subgroup analysis of CAL

#### Therapeutic strategies of CAL

A subgroup analysis of patients in the CAL cohort was performed. Patients were retrospectively grouped into three primary treatment groups. There was no difference in the age and sex distribution between cohorts. There was a statistically significant difference in the Clavien–Dindo grade, visualized in Table 2, and in the comprehensive complication index regarding the primary LAR (before treatment for CAL) between treatment groups. Group C also showed an already significantly elevated morbidity for their primary treatment before the occurrence of a CAL, compared to group A due to a higher comprehensive complication index (post hoc Bonferroni: CI:7.76–64.99, *p* = 0.009), but not in comparison to the interventional group (post hoc Bonferroni: CI:5.78–33.70, *p* = 0.252).

**Table 2:** Patient characteristics of the treatment groups

Factor*		Group A, *n* = 5	Group B, *n* = 22	Group C, *n* = 12	*p*-value
Age in years, mean (median, range)		62.4 (63.0, 46–73)	65.4 (66.5, 39–89)	59.7 (61.5, 48–68)	0.316
Sex (%)	Female Male	0 (0) 5 (100)	6 (27.3) 16 (72.7)	3 (25.0) 9 (75.0)	0.580
Clavien–Dindo at primary LAR (%)*	0 Grade I Grade II Grade III Grade IV	4 (75.0) 0 (0) 1 (25.0) 0 (0) 0 (0)	5 (22.7) 0 (0) 5 (22.7) 11 (50.0) 0 (0)	2 (16.7) 0 (0) 1 (8.3) 2 (16.7) 6 (50.0)	** < 0.001**
Therapeutic course (%)					
Multiple therapies	More than one therapy required Single therapy approach	3 (60.0) 2 (40.0)	14 (63.6) 8 (36.4)	9 (75.0) 3 (25.0)	0.799
Surgical revision	Surgical revision No surgical revision	3 (60.0) 2 (40.0)	9 (40.9) 13 (59.1)	6 (50.0) 6 (50.0)	0.732

*Some factors do not add up to 100% as data might have been inconclusive or unavailable

There was no difference between the therapy groups regarding the need for multiple therapy or surgical revision. Additionally, there was no significant correlation between the treatment groups and the therapeutic success (pain: 60.0 vs. 81.8 vs. 75.0%; bowel continuity: 40.0 vs. 50.0 vs. 58.3%), as shown in Table 3. In contrast, a significant increase in risk for patients with multiple therapies receiving a temporary or permanent stoma was observed (RR = 2.44; CI:1.37–4.35; *p* = 0.006). Patients with successful primary therapy, and therefore no need for surgical revision, had a higher rate of natural bowel continuity (RR = 7.71; CI:2.06–28.83; *p* < 0.001).

**Table 3:** Therapeutical outcome of the treatment groups

Factor		Group A, *n* = 5	Group B, *n* = 22	Group C, *n* = 12	*p*-value
Therapy success (%)					
Pain symptoms	No pain Pain	3 (60.0) 2 (40.0)	18 (81.8) 4 (18.2)	9 (75.0) 3 (25.0)	0.502
Bowel continuity	Continuity Stoma	2 (40.0) 3 (60.0)	11 (50.0) 11 (50.0)	7 (58.3) 5 (41.7)	0.819
Postoperative complications					
Comprehensive complication index (score), mean (range)		4.2 (0–21)	26.6 (0–85)	40.6 (0–66)	**0.011**

### Analysis of protective stoma reversal in patients with CAL

During the primary LAR, 25 patients (64.1%) of the CAL cohort (*n* = 39) received a protective ileostomy. Three (7.7%) patients already had a temporary stoma at the time of primary oncological operation, which was created preoperatively to treat an intestinal obstruction caused by the tumor, and 10 (25.6%) patients had neither a protective nor a temporary stoma at the time of the surgery. No data was documented for one patient. In the postoperative course of the 25 patients with protective loop ileostomy, 21 (84%) were reversed (Table 4).

**Table 4:** Patient characteristics of the stoma reversal subgroup analysis

Factor		No stoma reversal, *n* = 4	Reversal of protective stoma, *n* = 21	*p*-value
Age (years), mean (median, range)		72 (70.5, 66–81)	61.24 (63, 39–78)	0.068
Gender (%)	Female Male	0 (0) 4 (100)	3 (14.3) 18 (85.7)	1.000
Therapeutic course (%)				
Multiple therapies	More than one therapy required Single therapy approach	3 (75.0) 1 (25.0)	12 (57.1) 9 (42.9)	0.626
Surgical revision	(Re)Surgery No (re)surgery	2 (50.0) 2 (50.0)	13 (61.9) 8 (38.1)	1.000
Therapy success (%)				
Pain symptoms	Pain No pain	3 (75.0) 1 (25.0)	3 (14.3) 18 (85.7)	**0.031**
Bowel continuity	Continuity Stoma	0 (0) 4 (100)	14 (66.7) 7 (33.3)	**0.026**

**Fig. 2:**
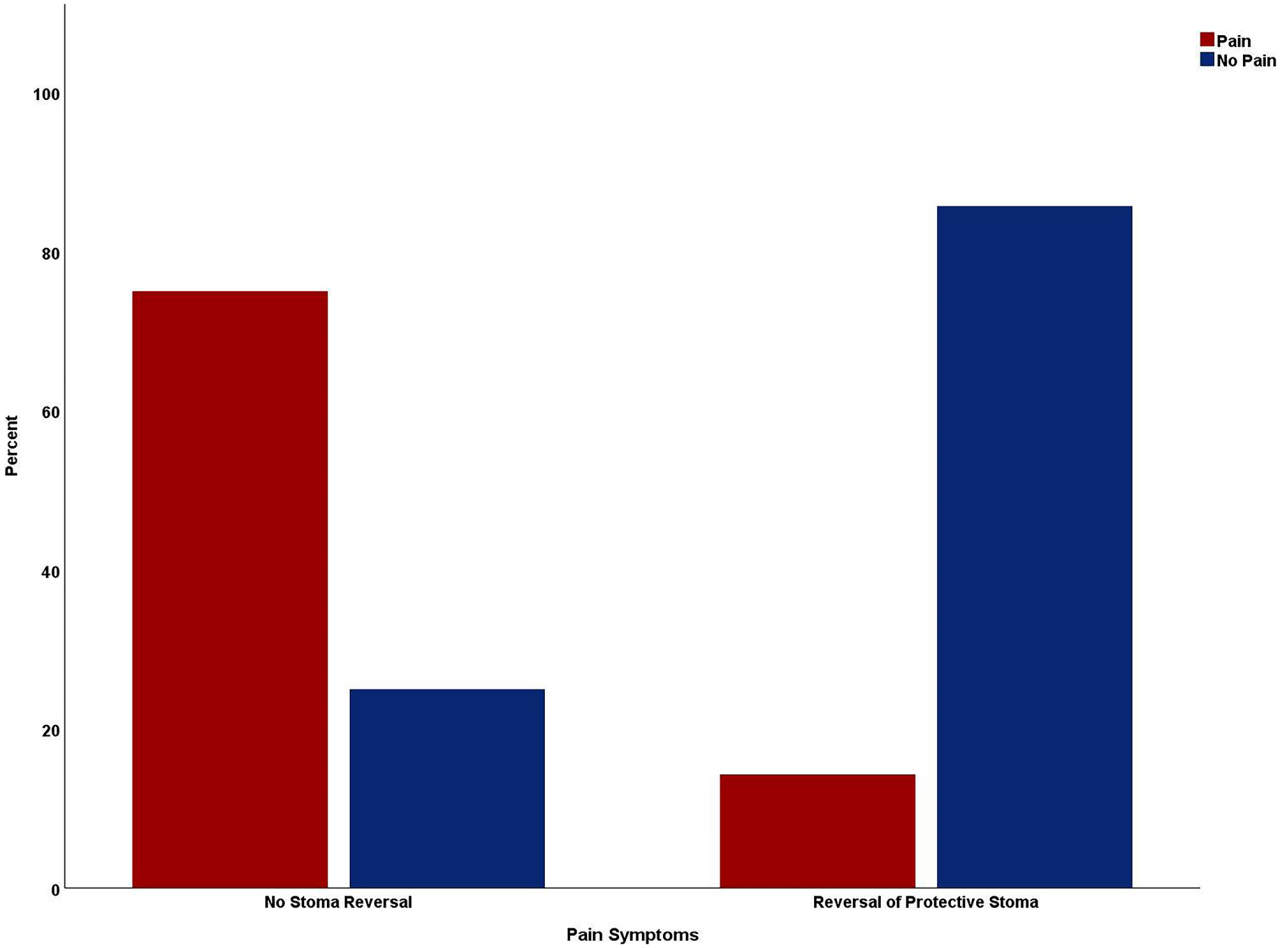
Comparison of the rate of post-therapeutic pain symptoms between subgroups

All stoma reversals proceeded without any intraoperative complications. There was a statistically significant association between stoma reversal and natural postoperative bowel continuity (RR = 3; CI:1.64–5.49; *p* = 0.026), meaning patients without stoma reversal had an increased risk of receiving a permanent stoma in the further therapy course. In the stoma reversal cohort, seven patients (33.3%) required some form of stoma (4 patients: discontinuity resection with the creation of a permanent stoma, three patients: temporary stoma) anyways. In addition, a statistically significant correlation between stoma reversal and less post-therapeutic pain symptoms (RR = 5.25; CI:1.60–17.27; *p* = 0.031) was observed, visualized in Fig. 2.

### Patient survival of the CAL cohort

**Fig. 3:**
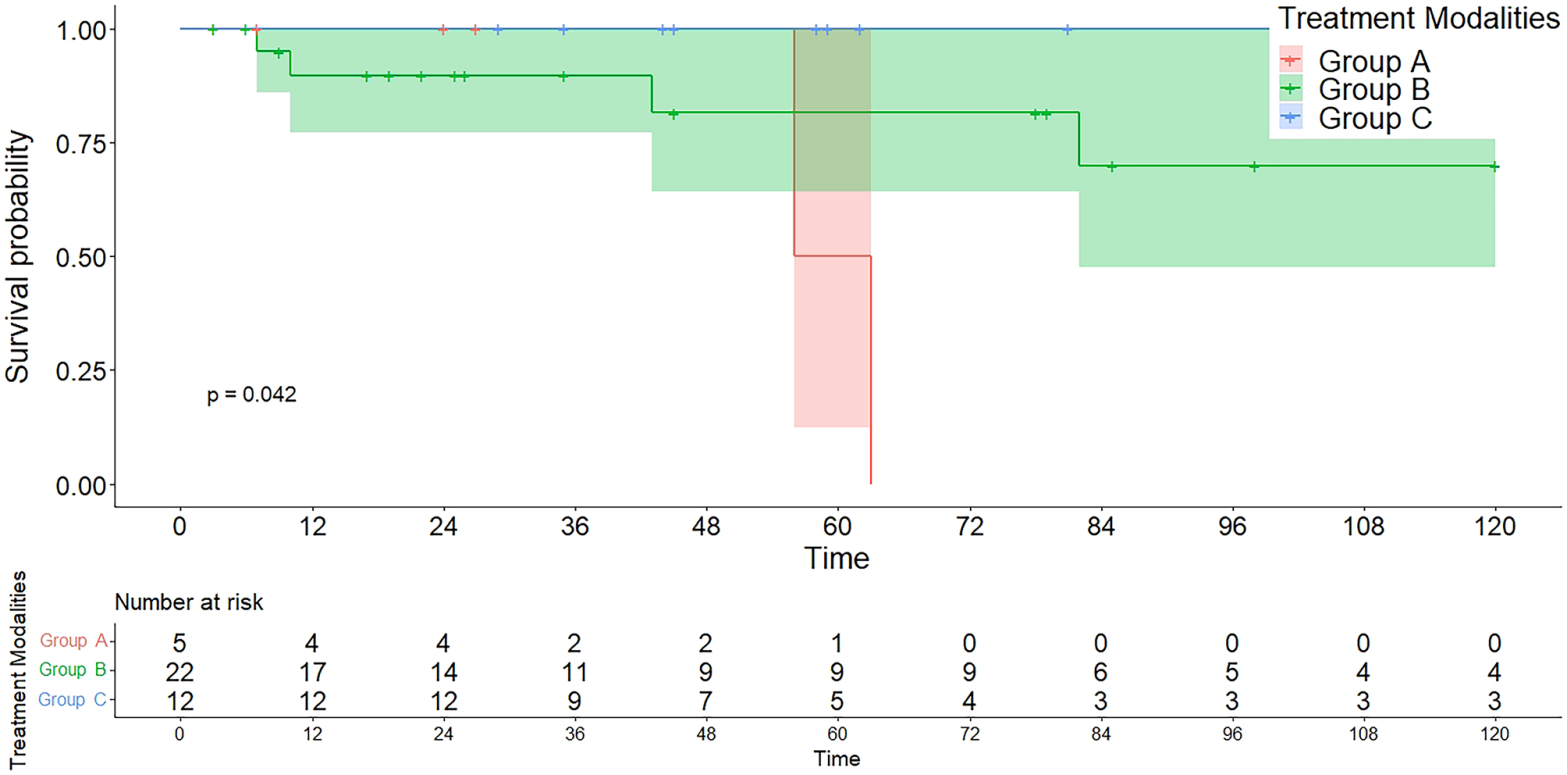
Ten-year survival with confidence intervals stratified by treatment modality

All 39 patients with CAL had either a valid final follow-up or a documented death. Eight patients (20.5%) of the study population had a reported death, and the mean overall survival was 163.3 months (median: 213 months). Survival analysis comparing treatment groups A/B/C showed that patients in group A had an average overall 10-year mean survival of 59.5 months, in contrast to 126.8 months in the interventional group and 192.0 months in the surgical group (log rank: *p* = 0.042; Breslow: *p* = 0.226), as can be seen in Fig. 3.

**Fig. 4:**
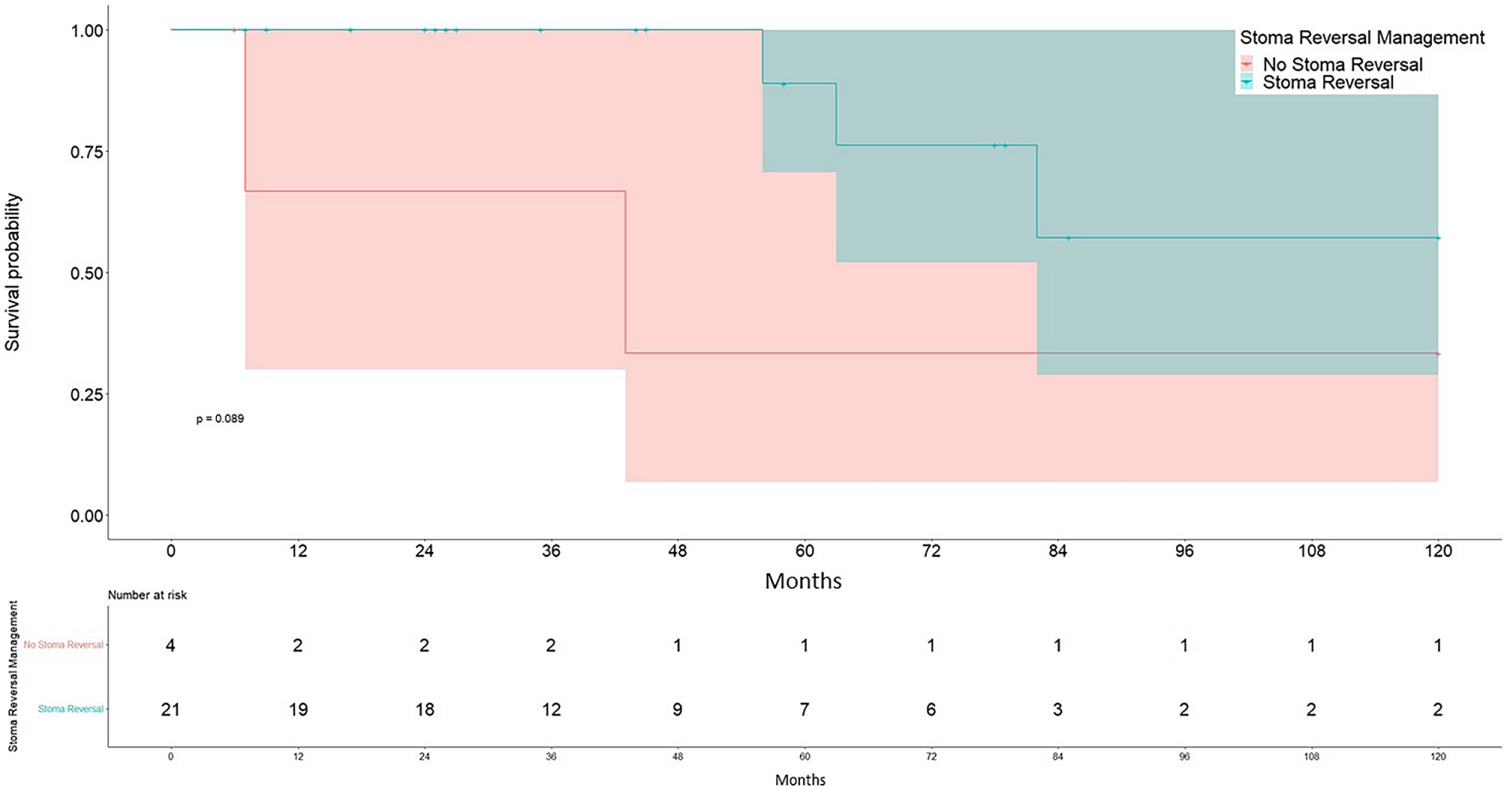
Ten-year survival with confidence intervals stratified by stoma reversal decision

Analysis of the overall 10-year survival in the stoma reversal group compared to the no stoma reversal group showed a significantly longer mean survival of 98.4 months in comparison to 56.7 months in the no-stoma reversal group (log rank: *p* = 0.089; Breslow: *p* = 0.004), as can be seen in Fig. 4.

## Discussion and conclusions

This study retrospectively analyzed the prevalence, therapy, and outcome of chronic anastomotic dehiscence following resection of low rectal cancer. In the study population, 71 patients (17.9%) developed any type of anastomotic leakage after LAR. According to recent literature, this rate is in the upper range, which might be attributed to patients with late-onset AL being added, and most studies only reporting on AL during the hospital stay [2, 3]. Moreover, patients primarily treated in other centers increased the rates of patients with CAL attended at our reference hospital. A total of 39 patients treated for CAL with persisting presacral sinus were further analyzed. This represents 54.9% of all considered anastomotic leakages and 9.8% of the total cohort and is therefore in accordance with the cross-sectional study by Borstlap et al. which reports rates of 48% and 9.5% [18].

Initial treatment modalities (conservative, interventional, or surgical) for CAL showed no influence on the rate of patients with chronic pain or the rate of reconstruction of bowel continuity. However, surgical therapy showed a significant advantage in patients’ survival compared to the other initial treatment modalities, despite a higher comprehensive complication index and Clavien–Dindo grade, at the time of low anterior resection, in the surgical group. Nevertheless, this survival benefit might be due to the small patient cohort and a selection bias, which was not analyzable through a retrospective study. The rate of surgical revisions and multitherapy between the initial interventional and surgical groups was comparable. This data should be seen as a trigger for future studies analyzing if primary surgical management should be considered the first option for CAL in patients presenting as fit for surgery because the limited sample size of this study does not allow for a therapy suggestion.

Factors influencing the outcome, such as multitherapy, secondary surgical revision, or stoma reversal, were evaluated in the further therapy course. A statistically significant negative relationship between multitherapy and bowel continuity was observed, wherein patients without multiple therapies had a higher rate of natural bowel continuity. Secondary surgical revision also correlated significantly with therapeutic success. Patients with secondary reoperation showed an increased rate of pain symptoms, while patients requiring no revision required a temporary or permanent stoma less often. These results again emphasize the possible association of primary surgical management to reduce the rate of unsuccessful treatment modalities. Subsequently, the influence of postoperative reversal of a protective loop ileostomy was examined, showing a significant coherence between stoma reversal and natural bowel continuity. Patients without reversal had an increased risk of staying with a permanent stoma. Although stoma reversal is naturally directly associated with bowel continuity, four (19%) patients nevertheless required a discontinuity resection to create a permanent stoma, and three (14%) patients required a temporary secondary stoma. In addition, there was a significant correlation between stoma reversal and post-therapeutic pain symptoms, showing that patients without reversal suffered from a higher risk of developing post-therapeutical pain. Patients with reversal also had a statistically significant overall 10-year survival advantage in this small cohort. According to this data, the influence of protective ileostomy reversal despite persistent CAL should be further assessed in larger cohorts, which might also be achieved in this specific cohort through multicentricity. One possible explanation for this suggested effect is that stoma reversal counteracts dehydration and electrolyte deficiency, thereby supporting granulation and epithelialization at the leakage and increasing its healing tendency [19]. The reversal of a protective stoma thus has a positive association with the therapy success regarding bowel continuity and symptomatic complaints, and survival. However, misinterpretation as a result of the small sample size and selection bias, due to preoperative assessment, is possible. The proposed selection bias is a possible confounder as some patients might not be fit enough for stoma reversal surgery or suffer from more severe comorbidities inhibiting stoma reversal.

Moreover, various clinical risk factors were analyzed. Hereby, neoadjuvant radiochemotherapy and neoadjuvant chemotherapy were identified as factors increasing the possibility of developing chronic leakages as already described in the literature.

## Limitations

This study was performed in a retrospective, non-randomized setting and had a small study population, especially in the subgroup analysis on stoma reversal. Accordingly, there might be a higher occurrence of type II errors. In addition, the analyzed period spans 25 years, during which different treatment concepts without constant therapeutic strategies as well as new surgical methods have been established. Furthermore, patients from peripheral hospitals influenced the study population through inconsistent therapy concepts and pre-existing complex courses. As a result, adjustment for confounding was not performed due to the distinct and limited sample size. Results should be interpreted accordingly. Although rectovesical and/or rectovaginal fistulas can be a possible clinical characteristics of CAL, these patients were excluded because they require different treatment modalities with involvement of additional departments. For future studies, patient-reported outcome should be considered an indicator for therapy success, as this allows a more detailed interpretation.

## Conclusion

Summarizing our results for therapeutical management, our data suggest that patients, which require a surgical revision or any kind of other additional therapy after their primary treatment, might suffer from reduced rates of bowel continuity. Nevertheless, before a specific treatment recommendation is possible, these results have to be validated in larger cohorts to examine if in patients with a CAL after LAR, primary surgical treatment should be preferred. In our cohort primary surgical treatment showed the same therapy result in the sense of bowel continuity and absence of pain. Furthermore, our data suggests that it may reduce the rate of patients receiving multiple therapies due to unsuccessful conservative or interventional treatment attempts.
